# Hospital-provision of essential primary care in 56 countries: determinants and quality 

**DOI:** 10.2471/BLT.19.245563

**Published:** 2020-08-27

**Authors:** Catherine Arsenault, Min Kyung Kim, Amit Aryal, Adama Faye, Jean Paul Joseph, Munir Kassa, Tizta Tilahun Degfie, Talhiya Yahya, Margaret E Kruk

**Affiliations:** a Harvard T.H. Chan School of Public Health, Harvard University, 665 Huntington Avenue, Boston, Massachusetts 02115, United States of America.; b Office of Member of Parliament, Gagan K Thapa, Kathmandu, Nepal.; c Faculté de Médecine, Département de Santé Publique, Université Cheikh Anta Diop, Dakar, Senegal.; d Hôpital Universitaire de Mirebalais, Zanmi Lasante, Haiti.; e Ministry of Health of Ethiopia, Addis Ababa, Ethiopia.; f Department of Reproductive Health and Population Studies, College of Medicine and Health Sciences, Bahir Dar University, Bahir Dar, Ethiopia.; g Quality Management Sub-unit, Ministry of Health, Community Development, Gender, Elderly and Children, Dodoma, United Republic of Tanzania.

## Abstract

**Objective:**

To estimate the use of hospitals for four essential primary care services offered in health centres in low- and middle-income countries and to explore differences in quality between hospitals and health centres.

**Methods:**

We extracted data from all demographic and health surveys conducted since 2010 on the type of facilities used for obtaining contraceptives, routine antenatal care and care for minor childhood diarrhoea and cough or fever. Using mixed-effects logistic regression models we assessed associations between hospital use and individual and country-level covariates. We assessed competence of care based on the receipt of essential clinical actions during visits. We also analysed three indicators of user experience from countries with available service provision assessment survey data.

**Findings:**

On average across 56 countries, public hospitals were used as the sole source of care by 16.9% of 126 012 women who obtained contraceptives, 23.1% of 418 236 women who received routine antenatal care, 19.9% of 47 677 children with diarrhoea and 18.5% of 82 082 children with fever or cough. Hospital use was more common in richer countries with higher expenditures on health per capita and among urban residents and wealthier, better-educated women. Antenatal care quality was higher in hospitals in 44 countries. In a subset of eight countries, people using hospitals tended to spend more, report more problems and be somewhat less satisfied with the care received.

**Conclusion:**

As countries work towards achieving ambitious health goals, they will need to assess care quality and user preferences to deliver effective primary care services that people want to use.

## Résumé

**Objectif:**

Évaluer l'utilisation des hôpitaux pour quatre services de soins de santé primaires essentiels dans plusieurs centres de santé communautaires situés dans des pays à faible et moyen revenu, et examiner les différences de qualité entre hôpitaux et centres de santé communautaires.

**Méthodes:**

Nous avons prélevé des données provenant de toutes les enquêtes démographiques et sanitaires menées depuis 2010 sur le type d'établissement fréquenté pour obtenir des moyens contraceptifs, des soins prénatals de routine ainsi qu'une prise en charge de la diarrhée d'importance mineure, la toux ou la fièvre chez l'enfant. Nous avons employé des modèles de régression logistique à effets mixtes pour examiner les associations entre le recours aux hôpitaux d'une part, et les covariables individuelles et nationales d'autre part. Nous avons mesuré le niveau de compétence des soins en nous fondant sur la réalisation d'actes cliniques essentiels lors des visites. Nous avons également analysé trois indicateurs d'expérience en tant que patient dans les pays où nous avons pu accéder aux résultats d'enquêtes d'évaluation des prestations de service.

**Résultats:**

Selon la moyenne établie sur 56 pays, les hôpitaux publics constituaient l'unique source de soins pour 16,9% des 126 012 femmes qui se sont procuré des contraceptifs, 23,1% des 418 236 femmes qui ont reçu des soins prénatals de routine, 19,9% des 47 677 enfants atteints de diarrhée et 18,5% des 82 082 enfants souffrant de fièvre ou de toux. Le recours aux hôpitaux était plus fréquent dans les pays plus riches où les dépenses en soins de santé par habitant étaient plus élevées, mais aussi parmi les citadines et les femmes plus aisées et mieux instruites. La qualité des soins prénatals était meilleure dans les hôpitaux de 44 pays. Dans un sous-groupe de huit pays, les gens qui se rendaient dans les hôpitaux avaient tendance à dépenser davantage, à signaler plus de problèmes et à se montrer moins satisfaits vis-à-vis des soins prodigués.

**Conclusion:**

À mesure que les pays s'efforceront d'atteindre des objectifs sanitaires ambitieux, ils devront évaluer la qualité des soins et les préférences des patients afin de proposer des services de soins de santé primaires efficaces auxquels les gens voudront faire appel.

## Resumen

**Objetivo:**

Estimar el uso de los hospitales para cuatro servicios esenciales de atención primaria que se ofrecen en los centros de salud de los países de ingresos bajos y medios, así como estudiar las diferencias de calidad entre los hospitales y los centros de salud.

**Métodos:**

Se obtuvieron los datos de todas las encuestas demográficas y de salud realizadas desde 2010 sobre el tipo de instalaciones que se usan para obtener anticonceptivos, atención prenatal de rutina y atención de la diarrea infantil leve y la tos o la fiebre. Se evaluaron las asociaciones entre el uso de los hospitales y las covariables a nivel individual y nacional mediante la aplicación de modelos de regresión logística de efectos mixtos. También se evaluó la competencia de la atención basada en la recepción de medidas clínicas esenciales durante las consultas. Asimismo, se analizaron tres indicadores de la experiencia de los usuarios de los países que disponían de datos de encuestas de evaluación de la prestación de servicios.

**Resultados:**

En un promedio de 56 países, los hospitales públicos fueron usados como la única fuente de atención por el 16,9 % de 126 012 mujeres que adquirieron anticonceptivos, el 23,1 % de 418 236 mujeres que recibieron atención prenatal de rutina, el 19,9 % de 47 677 niños con diarrea y el 18,5 % de 82 082 niños con fiebre o tos. El uso de los hospitales fue más común en los países más ricos con mayores gastos en salud per cápita y entre los residentes de las zonas urbanas y las mujeres más acaudaladas y mejor educadas. La calidad de la atención prenatal fue superior en los hospitales de 44 países. En un subconjunto de ocho países, las personas que acudieron a los hospitales tendían a gastar más, a reportar más quejas y a estar algo menos satisfechas con la atención recibida.

**Conclusión:**

A medida que los países se esfuerzan por alcanzar objetivos sanitarios ambiciosos, tendrán que evaluar la calidad de la atención y las preferencias de los usuarios para prestar los servicios de atención primaria efectivos que las personas pretenden usar.

## ملخص

الغرض تقدير الاعتماد على المستشفيات في خدمات الرعاية الأولية الأساسية التي تقدمها المراكز الصحية البلدان ذات الدخل المنخفض والمتوسط، واستكشاف الفروق في الجودة بين المستشفيات والمراكز الصحية.

الطريقة استخرجنا البيانات من جميع المسوح السكانية والصحية التي أجريت منذ عام 2010 حول نوع المرافق المستخدمة للحصول على وسائل منع الحمل، والرعاية الروتينية السابقة للولادة، ورعاية حالات الإسهال والسعال أو الحمى الطفيفة لدى الأطفال. وباستخدام نماذج التحوف اللوجيستي ذات التأثيرات المختلطة، قمنا بتقييم الارتباطات بين الاعتماد على المستشفى، والمتغيرات المشتركة على المستوى الفردي ومستوى البلد. قمنا بتقييم كفاءة الرعاية بناءً على فرص الحصول على الإجراءات السريرية الأساسية أثناء الزيارات. قمنا أيضًا بتحليل ثلاثة مؤشرات لتجربة المستخدم من البلدان التي يتوفر فيها بيانات مسح تقييم تقديم الخدمة.

النتائج في المتوسط ​​عبر 56 بلدًا، تم الاعتماد على المستشفيات العامة كمصدر وحيد للرعاية بواسطة 16.9% من 126012 امرأة حصلن على وسائل منع الحمل، و23.1% من 418236 امرأة حصلن على الرعاية الروتينية السابقة للولادة، و19.9% من 47677 طفلاً يعانون من الإسهال، و18.5% من 82082 طفلاً يعانون من الحمى أو السعال. كان الاعتماد على المستشفى أكثر شيوعًا في البلدان الأغنى مع ارتفاع نفقات الصحة للفرد، وبين سكان الحضر، والنساء الأكثر ثراءً والأفضل تعليمًا. كانت جودة الرعاية السابقة للولادة أعلى في المستشفيات في 44 بلدًا. في مجموعة فرعية من ثمانية بلدان، كان الأشخاص الذين يعتمدون على المستشفيات يميلون إلى إنفاق أكثر، والإبلاغ عن المزيد من المشكلات، ويكونون أقل رضا إلى حد ما عن الرعاية التي حصلوا عليها.

الاستنتاج بينما تعمل البلدان تجاه تحقيق أهداف صحية طموحة، فإنها سوف تحتاج إلى تقييم جودة الرعاية وتفضيلات المستخدم لتقديم خدمات رعاية أولية فعالة، يرغب الناس في الاعتماد عليها.

## 摘要

**目标:**

评估在中低收入国家使用医院来获取卫生中心提供的四类基本的初级护理服务的情况，并探讨医院和卫生中心之间的质量差异。

**方法:**

我们从 2020 年以来开展的所有人口统计和健康调查中提取出相关数据，这些调查涉及用于避孕、常规产前护理以及儿童轻微腹泻咳嗽或发烧护理的设施类型。利用混合效应逻辑回归模型，我们评估了医院使用与个人和国家层面协变量之间的相关性。我们根据就诊期间基本临床行动的接收情况来评估其护理能力。我们还分析了拥有现成的服务提供评估调查数据的国家的三个用户体验指标。

**结果:**

56 个国家平均而言，126012 名获取过避孕药具的女性中，仅选择公立医院的女性占 16.9％；418236 名接受常规产前护理的女性中，占比达 23.1％；47677 名腹泻儿童中，占比达 19.9％；82082 名发烧或咳嗽儿童中，占比达 18.5％。在人均卫生支出较高的富裕国家、城市居民以及较富裕受过良好教育的女性中，选择医院更为普遍。44 个国家的医院产前护理质量较高。其中八个国家，选择医院的人士往往花费更多，报告的问题更多，对所得到的护理也不太满意。

**结论:**

随着多国努力实现宏伟的卫生目标，它们将需要评估护理质量和用户偏好，以提供人们希望使用的有效的初级护理服务。

## Резюме

**Цель:**

Оценка использования больниц для оказания четырех основных услуг первичной медико-санитарной помощи, предлагаемых в медицинских центрах в странах с низким и средним уровнем доходов, а также изучение различий в качестве услуг между больницами и медицинскими центрами.

**Методы:**

Авторы извлекли данные из всех демографических и медицинских опросов, проведенных с 2010 года, о типах учреждений, используемых для получения противозачаточных средств, плановой дородовой помощи и лечения незначительных случаев детской диареи, кашля или лихорадки. Используя модели логистической регрессии со смешанными эффектами, авторы оценили связи между использованием больниц и ковариатами на индивидуальном и национальном уровнях. Авторы оценили компетентность оказания помощи на основе получения необходимых клинических действий во время обращений. Были также проанализированы три показателя опыта пользователей из стран, по которым имеются данные опроса по оценке оказания услуг.

**Результаты:**

В среднем в 56 странах государственные больницы использовались в качестве единственного источника медицинской помощи: 16,9% из 126 012 женщин, получавших противозачаточные средства, 23,1% из 418 236 женщин, получавших плановую дородовую помощь, 19,9% из 47 677 детей с диареей и 18,5% из 82 082 детей с лихорадкой или кашлем. Использование больниц было более распространенным явлением в более богатых странах с более высоким уровнем медицинских расходов на душу населения, а также среди городских жителей и более богатых, более образованных женщин. Качество дородовой помощи было выше в больницах в 44 странах. В подгруппе из восьми стран люди, обращающиеся в больницы, как правило, тратили больше, сообщали о большем количестве проблем и были несколько менее удовлетворены полученной помощью.

**Вывод:**

По мере работы стран над достижением амбициозных целей в области здравоохранения им необходимо будет оценивать качество медицинской помощи и предпочтения пользователей, чтобы предоставлять эффективные услуги первичной медико-санитарной помощи, которыми люди хотят пользоваться.

## Introduction

Achieving universal health coverage (UHC) will require affordable, high-quality primary care that is accessible to all people, at every age.^1^ Primary care is recognized as an essential platform for addressing the growing burden of chronic diseases and for detecting and managing infectious disease outbreaks in places that are most vulnerable to them.^2^^–^^5^ The 2008 World Health Report on primary health care emphasized that provision of high-quality primary care requires relocating the entry point to the health system from hospital outpatient departments to primary care centres.^6^ The more recent World Health Organization (WHO) global strategy on people-centred and integrated health services also calls for rebalancing health services towards primary care, and reducing the emphasis on the hospital sector.^7^ Primary care requires a relationship of trust between people and their providers.^6^ Settings such as busy hospital outpatient departments are not organized to build such relationships and produce people-centred care.^6^ In contrast, government health centres have usually been designed to work in close relationship with the community they serve, and can create the conditions for more comprehensive, person-centred continuing care.

Nonetheless, reports of people opting to use hospitals instead of health centres are common in low- and middle-income countries.^8^^–^^10^ Several factors may lead people to choose hospitals: negative perceptions about health centres (perceived poor quality or lack of trust), the convenience of hospitals, and the health policies in place.^11^^–^^13^ However, seeking care in hospitals may lead to excessive health-care spending and reduced equity and patient-centeredness and be a missed opportunity to promote relationships with primary care providers over time.^8^

Monitoring the proportion of people who use hospitals for essential health services and understanding the factors driving hospital use is important for strengthening primary care systems. In this analysis, we estimated the proportion of people who visited hospitals for four essential health services offered at health centres in low- and middle-income countries and explored the factors associated with hospital use. We also described differences in the quality of these services between hospitals and health centres.

## Methods

### Data sources

We used data from all demographic and health surveys conducted in low- and middle-income countries since 2010 and included the most recent survey available in each country (as of 20 January 2020). The demographic and health surveys are nationally representative household surveys that collect data on population health indicators with a strong focus on maternal and child health. Sampling strategies and methods have been described previously.^14^ We obtained data on country characteristics and purchasing power exchange rates from the World Bank’s world development indicators database^15^ and worldwide governance indicators project.^16^

We also included data from service provision assessment surveys. These surveys use nationally representative samples or censuses or near censuses of the country’s health facilities to provide a comprehensive overview of health service delivery in a country.^17^ We included the most recent surveys conducted in the same timeframe as the corresponding demographic and health survey (2010–2018) that included exit interviews with people attending family planning, antenatal care and sick-child care services.

### Essential health services

We described the type of health facility used for four non-urgent health services typically provided in primary care settings: (i) contraceptives, (ii) routine antenatal care, (iii) care for children younger than 5 years with non-severe diarrhoea; and (iv) care for children younger than 5 years with fever or a cough. These essential health services should be addressed in primary care settings and are four of the 16 tracer indicators selected to monitor progress towards UHC.^18^ In the demographic and health survey, women were asked to report the type of facility visited for each of the services. Those who used the public sector reported the level of the facility: whether it was a hospital or a lower-level facility dedicated to primary care such as a health centre or a clinic. For those who used the private sector, the demographic and health survey did not differentiate between hospitals and lower-level facilities. We therefore created three categories of facilities visited: (i) public hospitals (including district, regional, national and military hospitals); (ii) public health centres (including all non-hospital facilities); and (iii) any private health-care facilities. We excluded care received from homes, pharmacies, shops, drug sellers, traditional practitioners or a friend or relative.

To identify the usual or sole source of care and to exclude those people who may have been referred from a primary care facility to the hospital, we excluded anyone who reported using multiple types of facility for the same service. To restrict the sample to those with non-urgent and less severe conditions, we excluded women who were pregnant with twins or had previously had a perinatal death, as these women may be at greater risk of complicated pregnancies and may require more advanced antenatal care.^19^ For childhood diarrhoea, we excluded children who had blood in their stools. Among children with a fever or cough, we excluded those with suspected pneumonia as defined by the survey (a cough accompanied by short rapid breaths and difficulty breathing that is related to a problem in the chest). For contraceptives, we excluded women who used intrauterine devices, sterilization or implants as these more advanced methods may only be provided in hospitals.

To explore why people might visit public hospitals for services that are offered in primary care settings, we included a series of individual-level covariates available from the survey. These included urban residence, age group (15–19, 20–30, 31–40 or 41–65 years), secondary education, wealth quintiles and exposure to the media. We also explored associations with a series of country-level factors hypothesized to influence hospital use. These included year of the demographic and health survey (pre- or post-2015), the world region, country’s surface area, country’s total expenditure on health per capita, share of total health expenditure paid by patients out-of-pocket and an indicator of government effectiveness.^15^^,^^16^ We used country covariates for the year before the demographic and health survey. In cases where that year’s estimate was unavailable, we used the estimate for the closest year.

### Quality of care

To evaluate quality of care, we explored differences in provision of competent care and the user experience^12^ between public hospitals and health centres for the services included in this study.

We assessed competence of care in the demographic and health survey based on the receipt of essential clinical actions during visits. For antenatal care, we measured receipt of three items during consultations: blood pressure monitoring and urine and blood testing. For child diarrhoea, we assessed whether oral rehydration solutions were provided. For contraceptives, we measured whether women reported being counselled about potential side-effects when first prescribed the method, and being told about alternative contraception methods by the health provider. We were unable to identify any quality of care indicator for childhood fever or cough in the survey. These indicators offer only a limited view of the quality of these services. However, they are recommended as essential components of care according to WHO guidance^20^^–^^22^ and have been used by others to describe quality.^12^^,^^23^

We also analysed three indicators of user experience from a subset of eight countries with available data from service provision assessment surveys: cost of visit, number of problems experienced, and satisfaction. These indicators were measured during client exit interviews among those who sought family planning, antenatal care and sick child care services in public hospitals and in health centres. 

### Statistical analysis

First, we summarized the proportion of women seeking each of the four services in public hospitals, public health centres and in private facilities by country using individual-level sampling weights. We pooled the estimates across countries by weighting each country equally.

Second, to explore associations between individual and country-level factors and public hospital use for these services we used generalized linear mixed-effects models based on a logit-link function with a random intercept for the country. We repeated the models for each of the four health services. Because private sector users may differ from those seeking care in the public sector, the regression analyses were limited to those people who used public facilities. 

Finally, we compared quality of care in public hospitals and health centres using data from the demographic and health surveys and the service provision assessment surveys by estimating means for each indicator using individual-level (or client-level) sampling weights and weighting countries equally. 

We performed descriptive analyses using Stata version 16 (Stata Corp., College Station, United States of America) and fitted the mixed-effects models using R version 3.6.2 (R Foundation for Statistical Computing, Vienna, Austria).

## Results

A total of 58 countries conducted a demographic and health survey since 2010. However, Colombia and Turkey did not include data on care-seeking for sick children and were excluded. We, therefore, included 56 countries with surveys conducted from 2010 to 2018, the majority (31, 55.4%) conducted from 2015 to 2018.

### Essential health services

Data were available for 126 012 women who obtained contraceptives, 418 236 women who received antenatal care, 47 677 children younger than 5 years with diarrhoea and 82 082 children younger than 5 years with fever or cough. On average across the 56 countries, the proportions of women who sought care in public hospitals were 16.9% for contraceptives, 23.1% for antenatal care, 19.9% for childhood diarrhoea and 18.5% for childhood fever or cough (pooled averages weigh countries equally; country-specific averages use individual-level sampling weights; Table 1; available at: http://www.who.int/bulletin/volumes/98/11/19-245563). Public health centres were used for these services by 64.7%, 58.9%, 56.4% and 55.3% of women, respectively. The remaining women relied on the private sector.

**Table 1:** Type of facility used for services offered in primary care settings in 56 low- and middle-income countries

Country (year of survey)	Income group^a^	No. (%) of respondents using service																		
		Contraceptives					Antenatal care					Childhood diarrhoea					Childhood fever or cough			
		Total	Public hospitals	Public health centres	Private sector		Total	Public hospitals	Public health centres	Private sector		Total	Public hospitals	Public health centres	Private sector		Total	Public hospitals	Public health centres	Private sector
Afghanistan (2015)	Low	2 451	28.5	47.8	23.7		9 619	37.6	30.4	32.1		3 516	29.1	38.1	32.7		2 599	28.7	38.0	33.3
Albania (2017–2018)	Upper middle	40	13.6	80.0	6.4		1 704	58.6	24.3	17.1		94	39.3	52.7	7.9		122	30.6	66.2	3.2
Angola (2015)	Upper middle	746	35.2	57.4	7.4		6 576	31.4	60.6	8.1		863	31.3	56.8	11.9		872	30.9	59.8	9.4
Armenia (2016)	Lower middle	29	26.3	50.2	23.5		1 292	27.5	65.3	7.2		26	33.6	61.8	4.7		92	18.1	79.1	2.8
Bangladesh (2014)	Low	7 622	1.5	42.6	56.0		2 780	10.4	26.9	62.8		105	7.3	26.9	65.8		854	9.3	22.3	68.4
Benin (2017–2018)	Low	420	16.4	64.7	18.9		7 536	19.9	79.1	0.9		307	10.1	73.5	16.3		1 027	8.0	41.4	50.6
Burkina Faso (2010)	Low	1 326	14.3	81.5	4.2		9 406	6.4	92.3	1.4		838	17.4	79.6	3.1		1 609	16.7	79.6	3.6
Burundi (2017)	Low	1 494	9.1	85.9	5.0		7 929	8.5	77.1	14.4		1 504	5.4	81.8	12.8		2 903	4.8	81.3	13.9
Cambodia (2014)	Low	2 348	1.0	62.8	36.2		5 032	7.8	86.7	5.5		401	3.5	31.4	65.1		1 017	6.9	27.7	65.4
Cameroon (2011)	Lower middle	530	43.0	31.8	25.2		6 000	27.4	42.6	30.0		387	25.8	50.3	23.9		955	24.7	44.9	30.4
Chad (2014–2015)	Low	362	33.2	48.2	18.6		6 334	20.0	77.1	2.9		743	13.0	55.4	31.6		726	13.3	54.2	32.4
Comoros (2012)	Low	362	22.5	75.1	2.5		1 632	29.9	61.5	8.6		158	21.5	72.9	5.7		322	27.9	62.3	9.8
Congo (2012)	Lower middle	502	55.0	27.5	17.5		5 139	46.5	41.2	12.4		436	59.0	23.6	17.4		890	49.4	37.0	13.7
Côte d’Ivoire (2011–2012)	Lower middle	365	29.6	59.6	10.8		4 369	29.4	63.2	7.3		249	27.4	60.8	11.8		536	25.1	60.9	14.0
Democratic Republic of the Congo (2014)	Low	1 051	11.6	13.1	75.4		9 219	17.6	64.9	17.5		836	8.3	65.7	26.0		1 988	6.1	63.5	30.4
Dominican Republic (2013)	Upper middle	834	48.2	34.5	17.3		659	89.2	1.4	9.5		143	69.2	22.7	8.2		187	79.8	16.0	4.2
Egypt (2014)	Lower middle	2 690	7.3	88.7	4.0		12 701	2.1	9.8	88.2		1 090	9.5	14.3	76.2		1 940	8.9	13.4	77.7
Ethiopia (2016)	Low	2 583	2.3	81.1	16.5		4 326	6.1	88.2	5.8		534	4.8	71.3	23.9		371	5.2	63.9	30.9
Gabon (2012)	Upper middle	318	50.7	31.3	18.0		3 214	41.8	35.5	22.7		263	54.8	34.1	11.2		726	48.4	37.8	13.8
Gambia (2013)	Low	394	12.8	65.3	21.9		4 906	16.9	75.0	8.0		780	16.5	76.9	6.6		541	14.4	75.7	9.9
Ghana (2014)	Lower middle	628	29.7	61.4	8.9		3 675	48.4	40.5	11.1		233	29.3	54.8	15.9		466	32.7	50.0	17.4
Guatemala (2014–2015)	Lower middle	2 570	4.4	82.1	13.6		7 363	8.3	64.9	26.8		960	6.6	49.9	43.5		1 461	6.0	55.7	38.3
Guinea (2018)	Low	364	12.7	74.6	12.7		4 259	11.6	82.6	5.9		417	6.8	79.8	13.3		553	11.7	77.7	10.7
Haiti (2017)	Low	2 032	20.7	45.7	33.6		4 002	36.9	57.6	5.6		408	20.1	56.8	23.1		793	16.1	61.4	22.5
Honduras (2012)	Lower middle	3 001	4.4	79.0	16.6		7 399	14.3	68.8	17.0		824	16.5	63.2	20.2		1 523	12.5	64.4	23.1
India (2016)	Lower middle	19 799	13.5	24.7	61.8		108 798	33.2	27.8	39.0		12 908	14.4	8.3	77.4		23 211	16.1	9.1	74.8
Indonesia (2017)	Lower middle	11 926	0.2	34.1	65.7		10 525	2.6	33.1	64.3		1 238	2.4	42.2	55.4		3 128	1.7	38.1	60.2
Jordan (2017–2018)	Lower middle	1 129	4.7	73.5	21.9		5 937	19.0	12.3	68.7		404	21.3	29.5	49.2		468	13.8	39.3	46.9
Kenya (2014)	Low	6 652	18.1	50.3	31.6		6 206	31.8	52.0	16.3		1 362	17.7	63.4	18.9		3 355	17.7	60.3	22.1
Kyrgyzstan (2012)	Low	139	8.4	73.7	17.9		2 581	13.5	84.6	1.9		98	24.2	72.5	3.3		107	26.3	70.8	3.0
Lesotho (2014)	Lower middle	2 233	16.8	57.5	25.7		2 211	19.1	56.0	24.9		144	17.9	55.4	26.7		383	16.0	50.5	33.5
Liberia (2013)	Low	1 354	33.7	41.7	24.6		4 139	38.6	42.5	18.8		469	18.1	54.9	27.0		977	19.5	51.5	29.0
Malawi (2016)	Low	6 348	12.6	73.6	13.9		12 506	19.2	67.5	13.3		2 137	13.3	76.1	10.7		2 666	12.1	74.5	13.4
Maldives (2016–2017)	Upper middle	215	29.0	56.8	14.1		1 693	58.4	7.0	34.6		95	39.8	44.9	15.4		522	24.9	44.7	30.4
Mali (2018)	Low	592	2.0	78.0	20.0		4 863	2.3	91.5	6.2		407	1.8	80.8	17.4		427	0.7	81.5	17.8
Mozambique (2011)	Low	1 121	17.4	79.2	3.4		6 564	20.0	79.2	0.8		596	93.4	0.7	5.8		987	93.4	1.0	5.6
Myanmar (2015–2016)	Lower middle	2 204	10.7	68.3	21.1		2 304	28.3	59.0	12.7		207	24.4	47.1	28.5		437	18.8	47.9	33.4
Namibia (2013)	Upper middle	3 429	23.2	67.9	8.9		3 431	33.1	58.4	8.5		410	27.5	66.7	5.8		682	24.3	62.1	13.6
Nepal (2016)	Low	1 364	6.0	72.4	21.6		2 775	28.6	51.7	19.7		131	11.2	32.6	56.2		477	12.4	26.2	61.4
Nigeria (2018)	Lower middle	1 112	30.6	55.9	13.6		14 824	31.5	50.0	18.5		1 050	23.5	66.3	10.2		2 135	22.2	65.4	12.4
Pakistan (2017–2018)	Lower middle	580	19.0	61.3	19.7		5 160	23.8	2.6	73.7		1 143	14.0	2.9	83.1		1 870	12.8	3.6	83.6
Papua New Guinea (2016–2018)	Lower middle	1 228	19.5	74.6	6.0		4 902	24.9	70.2	4.9		476	18.8	75.0	6.2		695	16.2	78.8	5.0
Peru (2012)	Upper middle	3 665	12.2	85.2	2.7		5 924	20.3	71.0	8.8		299	20.9	62.7	16.4		1 392	16.7	58.8	24.5
Philippines (2017)	Lower middle	1 957	1.4	94.7	4.0		6 685	10.5	64.5	25.0		254	11.7	52.8	35.5		733	10.9	55.2	33.9
Rwanda (2014–2015)	Low	2 668	0.6	96.6	2.8		5 732	2.7	96.1	1.2		318	1.4	93.4	5.2		829	1.8	89.6	8.6
Senegal (2017)	Low	1 614	3.8	89.6	6.6		7 194	5.4	86.7	7.9		719	2.6	92.0	5.4		807	4.3	88.0	7.7
Sierra Leone (2013)	Low	2 058	15.0	69.7	15.2		7 365	17.4	79.6	2.9		564	16.4	77.9	5.7		1 615	12.9	82.9	4.2
South Africa (2016)	Upper middle	2 847	8.4	86.6	5.0		2 696	11.2	78.6	10.2		188	8.5	75.2	16.3		312	4.3	73.6	22.0
Tajikistan (2017)	Lower middle	337	9.9	90.1	0.1		3 761	6.6	92.7	0.7		404	16.3	80.3	3.5		245	17.3	76.6	6.1
Timor-Leste (2016)	Lower middle	1 084	11.5	83.7	4.8		3 990	15.2	82.2	2.6		474	13.1	79.1	7.9		481	15.1	77.7	7.2
Togo (2014)	Low	563	18.1	66.2	15.7		4 228	20.5	67.0	12.5		219	17.0	66.9	16.1		601	17.5	60.0	22.5
Uganda (2016)	Low	3 060	8.6	47.4	43.9		9 152	19.8	68.6	11.5		1 637	5.5	42.3	52.3		2 860	5.1	45.1	49.8
United Republic of Tanzania (2016)	Low	1 450	8.8	74.9	16.3		6 429	9.8	77.0	13.3		474	8.9	69.0	22.1		716	7.7	64.7	27.6
Yemen (2013)	Lower middle	1 765	28.5	52.4	19.2		5 638	31.9	19.3	48.9		1 246	25.4	30.7	43.9		1 161	24.6	29.8	45.6
Zambia (2013–2014)	Lower middle	3 481	7.0	88.1	4.9		8 667	10.9	84.5	4.7		1 087	8.7	86.2	5.1		2 317	7.6	86.8	5.6
Zimbabwe (2015)	Low	2 980	11.9	79.8	8.3		4 283	26.3	66.4	7.3		404	9.1	76.6	14.4		418	7.5	69.6	22.9
**All countries**	**NA**	**126 012**	**16.9**	**64.7**	**18.5**		**418 236**	**23.1**	**58.9**	**18.1**		**47 677**	**19.9**	**56.4**	**23.7**		**82 082**	**18.5**	**55.3**	**26.1**

NA: not applicable.

^a^ World Bank classification.^15^

Notes: Data shown include individual-level sampling weights. Public clinics include any non-hospital public facility type such as: primary or community health centres, welfare centres, health posts, public clinics, mobile clinics, government dispensaries, family welfare centres or public family doctor’s office.

**Fig. 1:**
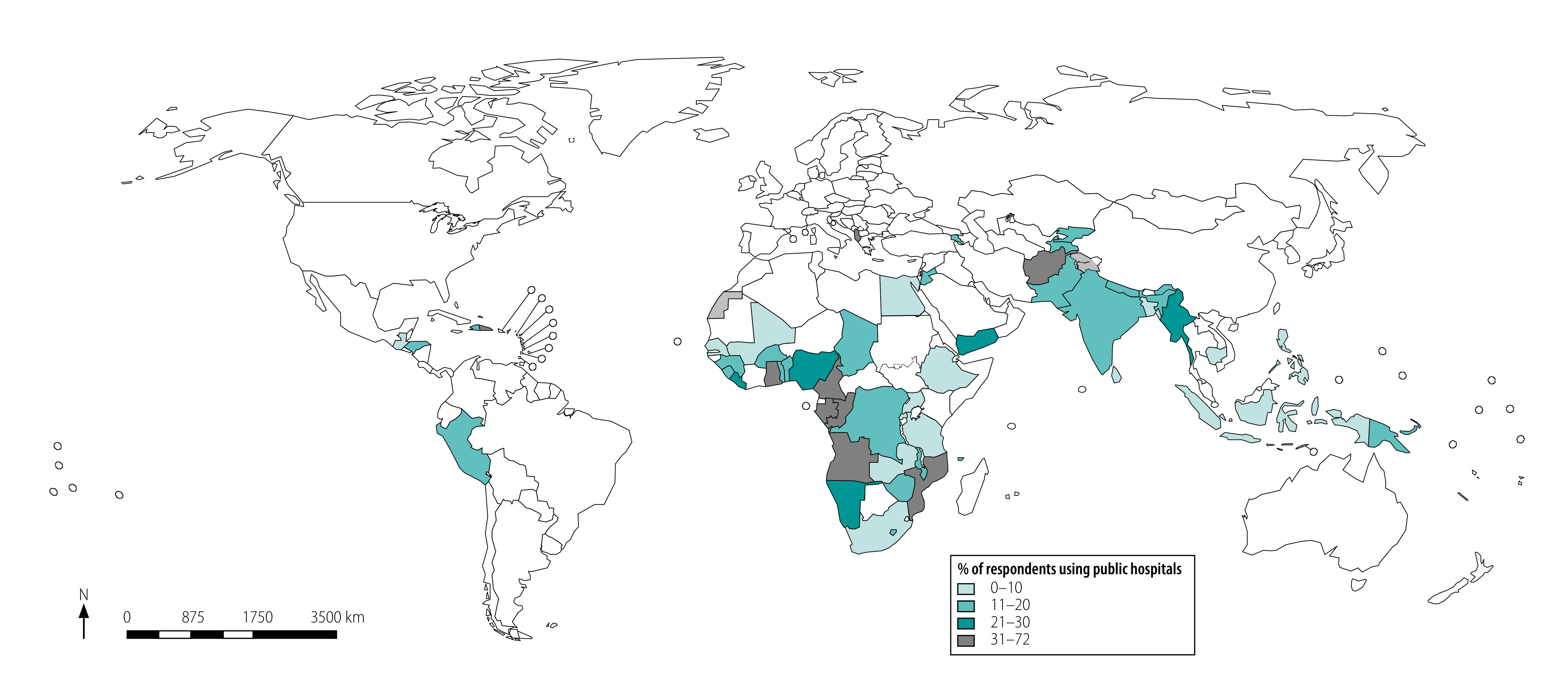
Use of public hospitals for four essential primary care services in 56 low- and middle-income countries

Notes: Average across four health services: contraceptives, antenatal care and care for child diarrhoea and child fever or cough.

Women in Cambodia, Ethiopia, Indonesia, Mali, Rwanda and Senegal had the lowest hospital use (less than 5% of women on average across the four services) while Albania, Congo, Dominican Republic, Gabon, Maldives and Mozambique had the highest use (more than 35% of women on average across the four services (Fig. 1 and data repository).^24^

**Fig. 2:**
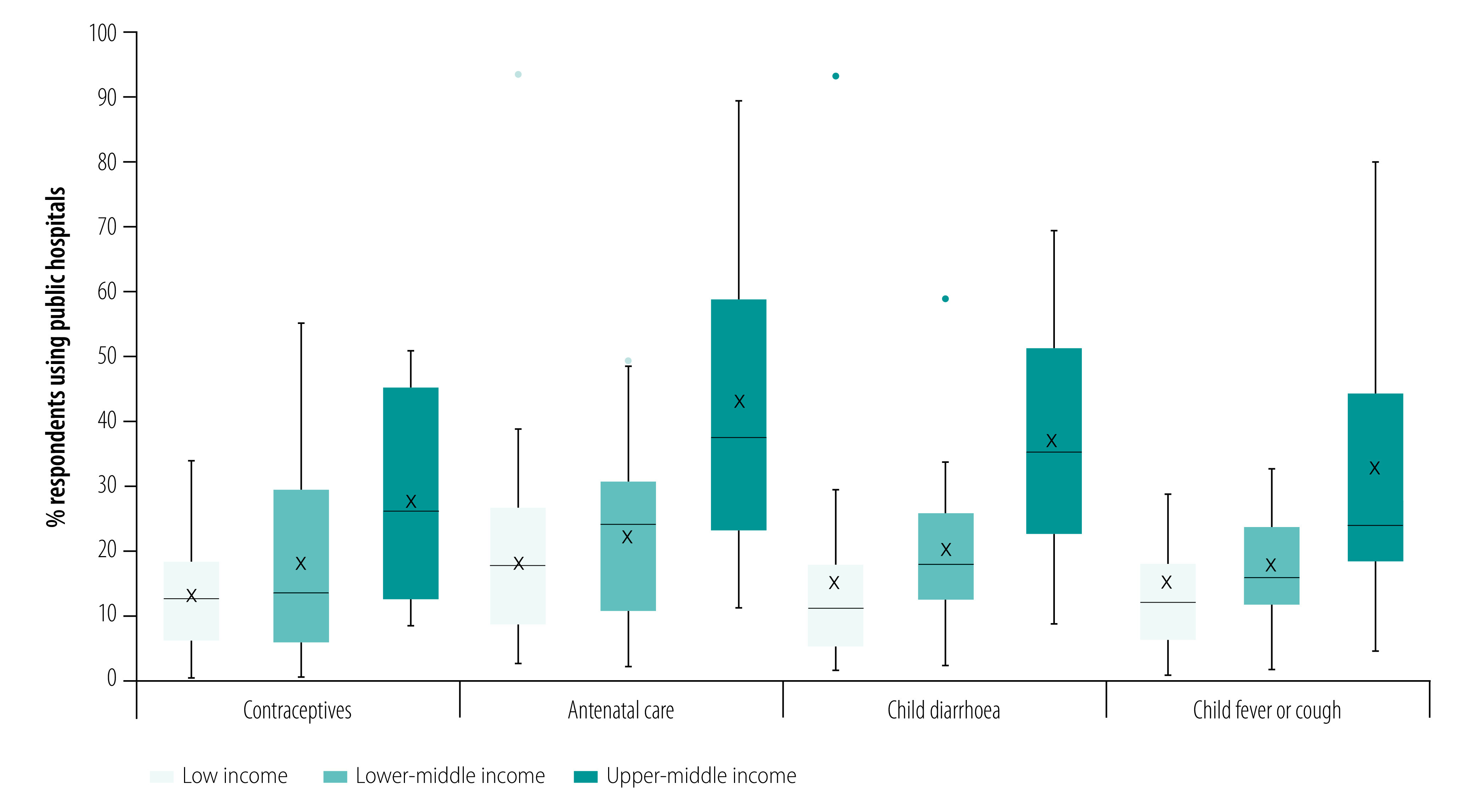
Use of public hospitals for four essential primary care services by country income group in 56 low- and middle-income countries

Notes: Boxes represent 25th percentile, median and 75th percentile. Mean is identified by x markers. Lines show upper and lower adjacent values, while dots are maximum outside values, as defined by Tukey et al., 1977.^25^

We found that the proportions of women using public hospitals for these four services tended to increase by country income group (Fig. 2). For example, an average of 43.0% of pregnant women used hospitals for routine antenatal care in upper-middle-income countries compared with 18.0% on average in low-income countries.

In all four regression models, we found that those visiting hospitals had a higher likelihood of living in urban areas, of being wealthier and of having a secondary education (Table 2). For example, mothers seeking medical advice or treatment for childhood diarrhoea in a hospital were twice as likely to belong to the wealthiest quintile than the poorest quintile (odds ratio, OR: 2.01; 95% confidence interval, CI: 1.77 to 2.29). We also found that women receiving antenatal care in hospitals were more likely to belong to an older age group and be regularly exposed to the media.

**Table 2:** Results of mixed-effects regression models for the associations between individual and country-level factors and public hospital use for four primary care services in 56 low- and middle-income countries

Variable	Contraceptives				Antenatal care				Care for childhood diarrhoea				Care for childhood fever or cough		
	No. of respondents	No. (%) using hospitals	OR (95% CI)		No. of respondents	No. (%) using hospitals	OR (95% CI)		No. of respondents	No. (%) using hospitals	OR (95% CI)		No. of respondents	No. (%) using hospitals	OR (95% CI)
**Individual characteristics**															
Area															
Urban	31 106	8 857 (28.5)	2.45 (2.34 to 2.56)		100 715	44 319 (44.0)	1.96 (1.92 to 2.00)		8 549	3 938 (46.1)	3.15 (2.91 to 3.40)		14 750	6 574 (44.6)	3.04 (2.87 to 3.23)
Rural	59 174	7 207 (12.2)	Ref.		226 892	60 146 (26.5)	Ref.		21 364	4 902 (23.0)	Ref.		35 791	7 923 (22.1)	Ref.
Wealth quintiles															
Q1 poorest	19 218	1 876 (9.8)	Ref.		78 375	17 656 (22.5)	Ref.		7 846	1 647 (21.0)	Ref.		12 817	2 521 (19.7)	Ref.
Q2	19 937	2 462 (12.4)	1.11 (1.03 to 1.19)		75 056	20 984 (28.0)	1.28 (1.25 to 1.32)		6 998	1 754 (25.1)	1.14 (1.04 to 1.25)		12 018	2 925 (24.3)	1.16 (1.08 to 1.24)
Q3	19 221	3 191 (16.6)	1.34 (1.25 to 1.43)		68 657	22 249 (32.4)	1.52 (1.48 to 1.56)		6 265	1 872 (29.9)	1.34 (1.22 to 1.48)		10 595	3 045 (28.7)	1.29 (1.19 to 1.39)
Q4	17 631	4 045 (22.9)	1.59 (1.48 to 1.71)		60 216	22 876 (38.0)	1.84 (1.79 to 1.90)		5 284	1 894 (35.8)	1.53 (1.38 to 1.70)		8 999	3 243 (36.0)	1.52 (1.40 to 1.65)
Q5 richest	14 273	4 490 (31.5)	1.84 (1.70 to 1.99)		45 303	20 700 (45.7)	2.45 (2.36 to 2.54)		3 520	1 673 (47.5)	2.01 (1.77 to 2.29)		6 112	2 763 (45.2)	1.97 (1.78 to 2.17)
Woman's age, years															
15–19	5 453	1 001 (18.4)	Ref.		20 833	5 621 (27.0)	Ref.		2 115	563 (26.6)	Ref.		3 251	871 (26.8)	Ref.
20–30	42 139	7 724 (18.3)	0.95 (0.87 to 1.03)		195 174	65 995 (33.8)	1.02 (0.99 to 1.06)		18 805	5 924 (31.5)	1.06 (0.94 to 1.20)		30 233	9 297 (30.8)	1.02 (0.92 to 1.13)
31–40	31 698	5 483 (17.3)	0.96 (0.88 to 1.04)		93 228	28 195 (30.2)	1.06 (1.02 to 1.10)		7 670	2 031 (26.5)	1.00 (0.88 to 1.15)		14 487	3 776 (26.1)	1.01 (0.91 to 1.12)
41–65	10 990	1 856 (16.9)	1.06 (0.96 to 1.16)		18 372	4 654 (25.3)	1.10 (1.04 to 1.16)		1 323	322 (24.3)	1.01 (0.84 to 1.22)		2 570	553 (21.5)	0.98 (0.85 to 1.13)
Any secondary education															
Yes	39 963	8 482 (21.2)	1.13 (1.08 to 1.18)		130 398	54 824 (42.0)	1.25 (1.22 to 1.27)		9 541	3 688 (38.7)	1.16 (1.07 to 1.25)		18 512	7 010 (37.9)	1.20 (1.14 to 1.27)
No	50 317	7 582 (15.1)	Ref.		197 209	49 641 (25.2)	Ref.		20 372	5 152 (25.3)	Ref.		32 029	7 487 (23.4)	Ref.
Media exposure^a^															
Yes	60 070	11 562 (19.2)	1.00 (0.95 to 1.05)		190 526	70 264 (36.9)	1.11 (1.09 to 1.13)		16 904	5 728 (33.9)	1.01 (0.95 to 1.09)		29 854	9 797 (32.8)	1.00 (0.95 to 1.06)
No	30 210	4 502 (14.9)	Ref.		137 081	34 201 (24.9)	Ref.		13 009	3 112 (23.9)	Ref.		20 687	4 700 (22.7)	Ref.
**Country characteristics**															
Surveyed post-2015															
Yes	51 548	8 664 (16.8)	0.65 (0.38 to 1.09)		219 075	75 892 (34.6)	0.67 (0.35 to 1.27)		19 198	5 725 (29.8)	0.40 (0.20 to 0.79)		28 940	8 578 (29.6)	0.34 (0.17 to 0.66)
No	38 732	7 400 (19.1)	Ref.		108 532	28 573 (26.3)	Ref.		10 715	3 115 (29.1)	Ref.		21 601	5 919 (27.4)	Ref.
Land area, millions km^2^	90 280	NA	1.03 (0.66 to 1.59)		327 607	NA	0.74 (0.43 to 1.28)		29 913	NA	1.14 (0.64 to 2.03)		50 541	NA	1.05 (0.59 to 1.86)
Government effectiveness index^b^	90 280	NA	0.36 (0.18 to 0.70)		327 607	NA	0.71 (0.30 to 1.67)		29 913	NA	0.82 (0.34 to 2.02)		50 541	NA	0.83 (0.33 to 2.05)
Total health expenditure per capita, hundreds int. $^c^	90 280	NA	1.17 (1.02 to 1.34)		327 607	NA	1.31 (1.11 to 1.55)		29 913	NA	1.10 (0.92 to 1.32)		50 541	NA	1.06 (0.89 to 1.26)
Share of out-of-pocket expenditure on health, %	90 280	NA	0.95 (0.81 to 1.11)		327 607	NA	1.05 (0.86 to 1.27)		29 913	NA	0.97 (0.79 to 1.19)		50 541	NA	0.99 (0.81 to 1.21)
Region^d^															
East African	26 020	4 218 (16.2)	Ref.		65 321	13 287 (20.3)	Ref.		8 340	1 708 (20.5)	Ref.		14 251	2 924 (20.5)	Ref.
Eastern Mediterranean	7 338	1 751 (23.9)	1.64 (0.55 to 4.89)		14 658	8 537 (58.2)	3.48 (0.90 to 13.55)		3 566	1 750 (49.1)	4.51 (1.09 to 18.69)		3 112	1 508 (48.5)	3.59 (0.87 to 14.84)
European	529	59 (11.2)	1.06 (0.28 to 3.93)		8 899	2 246 (25.2)	0.41 (0.08 to 2.05)		587	144 (24.5)	1.44 (0.27 to 7.83)		534	139 (26.0)	1.05 (0.20 to 5.64)
Middle African	1 988	1 098 (55.2)	4.63 (1.69 to 12.69)		31 789	11 549 (36.3)	2.10 (0.60 to 7.42)		2 826	1 025 (36.3)	1.45 (0.39 to 5.45)		5 048	1 744 (34.6)	1.18 (0.32 to 4.38)
Americas	11 596	1 642 (14.2)	0.82 (0.29 to 2.34)		22 349	4 832 (21.6)	1.15 (0.30 to 4.39)		2 041	394 (19.3)	0.99 (0.24 to 4.03)		4 298	736 (17.1)	0.89 (0.22 to 3.59)
Southern African	7 845	1 498 (19.1)	0.98 (0.23 to 4.21)		7 430	1 742 (23.5)	0.25 (0.04 to 1.43)		655	151 (23.0)	0.42 (0.07 to 2.63)		1 089	242 (22.2)	0.35 (0.06 to 2.11)
South East Asia	20 520	3 508 (17.1)	0.80 (0.30 to 2.12)		91 325	45 083 (49.4)	1.45 (0.43 to 4.88)		5 005	2 476 (49.5)	1.50 (0.42 to 5.44)		10 290	4 959 (48.2)	1.49 (0.41 to 5.39)
Western African	9 423	1 900 (20.2)	1.27 (0.57 to 2.84)		70 854	14 739 (20.8)	0.67 (0.25 to 1.81)		6 014	964 (16.0)	0.56 (0.20 to 1.60)		10 254	1 891 (18.4)	0.53 (0.19 to 1.49)
Western Pacific	5 021	390 (7.8)	0.56 (0.18 to 1.77)		14 982	2 450 (16.4)	0.53 (0.12 to 2.34)		879	228 (25.9)	1.03 (0.22 to 4.90)		1 665	354 (21.3)	1.06 (0.23 to 4.96)
**Analysis**															
Intercept	NA	NA	0.04 (0.02 to 0.10)		NA	NA	0.07 (0.02 to 0.23)		NA	NA	0.16 (0.05 to 0.51)		NA	NA	0.19 (0.06 to 0.64)
Variance estimate, null model	NA	NA	1.53 (−0.90 to 3.95)		NA	NA	1.81 (−0.83 to 4.45)		NA	NA	1.71 (−0.85 to 4.28)		NA	NA	1.64 (−0.87 to 4.14)
Variance estimate, full model	NA	NA	0.80 (−0.95 to 2.55)		NA	NA	1.27 (−0.94 to 3.47)		NA	NA	1.38 (−0.92 to 3.68)		NA	NA	1.35 (−0.93 to 3.62)

CI: confidence interval; Int. $: international dollar; NA: not applicable; OR: odds ratio; Ref.: reference category.

^a^ Women were categorized as being exposed to the media if they reported doing at least one of the following every week: reading newspapers or magazines, listening to the radio or watching television.

^b^ Government effectiveness index is estimated by the World Bank’s worldwide governance indicators project^16^ and is expressed in units of a standard normal distribution with a mean of zero and a standard deviation of one with higher values corresponding to better effectiveness.

^c^ Total health expenditures per capita were obtained from the World Bank’s world development indicators database^15^ and are expressed in hundreds of international $.

^d^ World regions were based on World Health Organization classifications and the African Region was divided into four sub-regions based on the United Nations Statistics Division classification.^26^

Notes: For each health service, we first calculated a null model with no covariates and only a country-specific random effect to model between-country variation in public hospital use.

Among country characteristics, we found that women visiting hospitals to obtain contraceptives and receive antenatal care had a higher likelihood of living in countries with higher health expenditures per capita (OR: 1.17; 95% CI: 1.02 to 1.34 and OR: 1.31; 95% CI: 1.11 to 1.55, respectively). Women visiting hospitals to obtain contraceptives were much less likely to live in countries with effective governments (OR: 0.36; 95% CI: 0.18 to 0.70). Women choosing hospitals for treatment of child diarrhoea and fever or cough were less likely to live in countries surveyed post-2015, indicating a potential reduction in hospital use over time.

### Quality of care

**Fig. 3:**
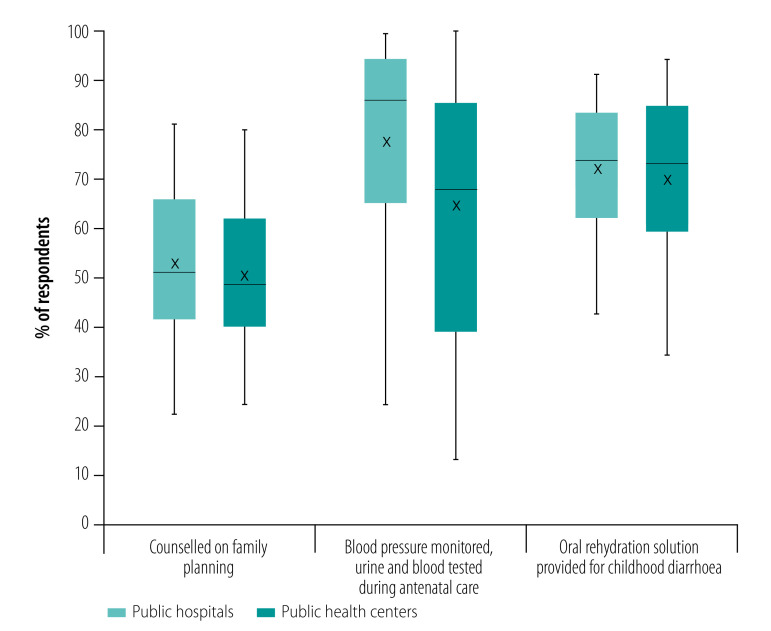
Differences in quality of family planning, antenatal care and sick child care between public hospitals and health centres in 56 low- and middle-income countries

Notes: Boxes represent 25th percentile, median and 75th percentile. Mean is identified by x markers. Lines show upper and lower adjacent values, while dots are maximum outside values as defined by Tukey et al., 1977.^25^

We found that, on average, women who received antenatal care in hospitals were much more likely to report having their blood pressure monitored and urine and blood samples taken compared with women who received antenatal care in health centres (Fig. 3). The differences were statistically significant in 44 of the 56 countries (*P* < 0.05; data repository).^24^ Only two countries (Albania and Tajikistan) had higher antenatal care quality in health centres. There were small differences in quality for the other two services, whereby women using hospitals were slightly more likely to report appropriate counselling when obtaining contraceptives or being provided with oral rehydration solutions for their child’s diarrhoea (statistically significant differences in 11 countries each; data repository).^24^

Data were available from service provision assessment surveys in eight low-income countries (Table 3). We found that those receiving care in hospitals spent more than those who visited health centres – an average of international dollars 1.09 more per visit. Costs were significantly higher in hospitals in seven of the eight countries (data repository).^24^ Although the number of problems reported was low overall, people using hospitals tended to report more problems on average than users of health centres. Differences were statistically significant in five countries. For example, hospital users were more likely to report experiencing problems with the amount of explanation received from their provider and with their ability to discuss concerns. In addition, in health centres, 81.3% of people reported being very satisfied with the services received, compared with 74.7% in hospitals. Differences in satisfaction were statistically significant in six countries (data repository).^24^

**Table 3:** Costs and experiences of care between users of public hospitals and health centres in eight low-income countries

Facility used	No. of respondents		Mean (SD)		
			Total paid for services, int. $^a^	Average no. of problems reported^b^	Very satisfied with services, %^c^
Public hospitals	11 224		2.24 (1.98)	1.5 (0.5)	74.7 (17.7)
Public health centres	21 564		1.15 (1.16)	1.2 (0.4)	81.3 (16.2)

Int. $: international dollar; SD: standard deviation.

^a^ The total paid for services are in international dollars converted using the purchasing power parity conversion rate for the year of the survey.

^b^ Average number of problems reported among the following 11 common problems: wait time, ability to discuss concerns with health worker, amount of explanation provided, visual privacy, auditory privacy, medication availability, hours services are provided, days services are provided, cleanliness of facility, staff treatment and cost of services. We calculated averages using individual sampling weights and countries were weighted equally.

^c^ Satisfaction was measured by the proportion of people who reported being “very satisfied with the services received” as opposed to “more or less satisfied” or “not satisfied.”

Notes: We extracted data from service provision assessment surveys conducted in Democratic Republic of the Congo 2018, Ethiopia 2014, Haiti 2017, Kenya 2010, Malawi 2013, Nepal 2015, Senegal 2017 and United Republic of Tanzania 2015 (see also the data repository).^24^

## Discussion

Using nationally representative surveys from 56 countries, we found that using hospitals for essential primary care services is relatively common in low- and middle-income countries. Around one in five people seeking contraceptives, routine antenatal care or care for minor childhood illnesses went to a public hospital instead of a health centre.

Using hospitals for these services was more common among the wealthiest, urban residents and the most educated women, reflecting that hospital use is highly inequitable. This finding may also reflect a lack of trust and a perception that the quality of care is poor in public health centres.^27^ An increasing number of studies are showing that people are willing to travel further distances or pay more out-of-pocket to seek what they consider better quality care.^28^^–^^31^ Rising expectations among wealthier populations may lead people aspiring to higher standards of care to bypass health centres, believing that quality is better at hospitals.^10^ In low- and middle-income countries, hospitals tend to be substantially better equipped, have better diagnostic and laboratory capacity and employ a greater number of physicians and qualified health providers than health centres.^32^ Hospitals may therefore be seen as a more effective solution to primary care needs. These aspects may especially attract those who can afford to visit hospitals.

In adjusted models, we found that a country’s insurance model (proportion of total health spending that was out-of-pocket) did not influence the source of care. However, the positive association of hospital use with a country’s total health expenditure per capita may reflect that governments are disproportionately directing health resources to hospitals, by building more, investing in quality, or both. The negative association between government effectiveness and people’s use of hospitals for essential health services may capture countries’ ability to successfully manage a larger set of public health facilities, with resulting better services in health centres. Use of hospitals for children with minor illnesses was less likely in countries surveyed post-2015. Inferences related to changes in hospital use over time must be interpreted with caution as these data are cross-sectional and we only included one survey per country.

Our sub-analysis on quality of care showed that in many countries women who attend antenatal care in hospitals are more likely than those in health centres to have their blood pressure monitored and urine and blood tested. This finding may be linked to a lack of diagnostic capacity for urine and blood testing in health centres or poorer competence levels of providers. In some countries, those who sought care in hospitals for childhood diarrhoea were more likely to receive oral rehydration solutions. This result is surprising given that oral rehydration is a simple low-cost intervention with important health benefits that should be easily provided in health centres.^33^ Our sub-analysis in eight countries showed that hospital users spent nearly twice as much for the same services. This finding reflects greater spending on some combination of provider or booking charges, diagnostic services and prescriptions. The cost differential is likely an underestimate, because travel costs, which are likely to be higher for hospital visits, were not included.^34^^,^^35^

Our findings are consistent with other studies in low- and middle-income settings. In Ethiopia, the national health accounts survey estimated that around 17% of all outpatient care was provided in government hospitals and that urban residents were three times more likely to use hospitals for outpatient services than were rural residents.^36^ In contrast, rural residents were more likely to attend health centres. In six middle-income Latin American and Caribbean countries, half of respondents had used hospital emergency departments for a condition they considered treatable in primary care in the past 2 years.^37^ In high-income countries, studies showed that use of hospitals for non-urgent care was generally more common among low-income individuals and those without health insurance.^38^^,^^39^ Use of hospital emergency departments as a usual source of care has been often studied in high-income countries, and is widely recognized as problematic given the higher cost and lack of continuity of care.^40^^,^^41^ Availability of a source of care that performs primary care functions well is associated with more effective, equitable and efficient health services and better overall health for individuals.^41^^,^^42^

Our study covered a large set of lower-income countries using a standardized measurement approach and can therefore provide input for future planning of health systems in low- and middle-income countries. Nonetheless, our study has limitations. First, because the demographic and health surveys do not include specific hospital and clinic categories for the private sector, we were unable to include private hospitals, which likely account for a considerable share of care-seeking in many countries. Second, our analysis was limited to reproductive, maternal and child health services. Even larger proportions of people may be using hospitals for routine care for diabetes, hypertension or human immunodeficiency virus infection, as these services have more recently been added to essential packages in low- and middle-income countries.^43^ In addition, because of data limitations, our analysis only included women (aged 15–49 years) and children younger than 5 years, and did not include data on primary care services for teenagers or adult men. The true proportion of hospital use for the full range of services offered in health centres is likely considerably higher if other health services and private sector hospitals were included. Facility types and disease severity were also self-reported by women interviewed in demographic and health surveys and may be misclassified. Our regression analyses excluded other factors potentially affecting the magnitude of hospital use, including the type of insurance cover as well as the relative share of private sector facilities. Future research should analyse care-seeking patterns in the light of these potentially confounding factors. Finally, coefficients from multilevel logistic regressions have a conditional, within-cluster interpretation and the magnitude of the association between outcomes and country covariates must be done with caution.^44^

Our findings have important implications for the design of health systems in low- and middle-income countries and for improving health outcomes. WHO guidance recommends community health centres for provision of people-centred primary care.^6^^,^^45^ Despite this recommendation, we found that many users select hospitals for four services that should be routinely provided with good quality at lower-level facilities. The comprehensive, coordinated, continuous, person-centred care and accessible services that are the hallmark of high-quality primary care may be difficult to provide in hospitals that are geared to more episodic care.^46^ The services will also almost certainly be more expensive.

Based on these findings, we identify three policy implications. First, the roles of the different levels of care in low- and middle-income country health systems need to be clearly defined. The *Lancet Global Health* Commission on high-quality health systems in the sustainable development goals era recommended redesigning health systems to ensure that the right health services are provided by the right provider, working in the right place in the health system.^12^ Health system structures and facility roles are often poorly defined in low- and middle-income countries. Many types of public health facilities, from health posts to regional hospitals, are expected to provide the full range of essential primary care services, including family planning, antenatal care, child vaccination and routine chronic disease management. Many hospitals in low- and middle-income countries have outpatient departments dedicated to these services. Meanwhile, some hospitals struggle to provide high-quality emergency and surgical care and to save the lives of those with complex injuries, obstetric complications or illnesses.^47^^–^^49^ In some countries, higher-level hospitals have become overcrowded, while primary care facilities remain underutilized.^50^ Reorganizing health-service delivery could improve health outcomes and patient confidence by allowing facilities and providers to focus on the services that they are geared towards providing. The role of the private sector should also be considered in planning service delivery.

Second, if people are to be redirected to use health centres for primary care services, there need to be improvements in the competence, comprehensiveness and convenience of care in health centres, including access to diagnostic services and appropriate opening hours. Governments must ensure that patients in health centres receive the core diagnostic services, treatments and counselling they need to maintain and improve their health. These reforms are needed to improve people’s trust in health centres so that they are willing to use them. 

Third, if hospitals are to continue providing a substantial proportion of these primary care services, they need to make improvements to the user experience, people-centredness and continuity and integration of care, while costs must be reduced to ensure equitable access.

To stop the drift towards use of hospitals, structural health system investments such as a strong primary care workforce, excellent management and well-equipped health centres that operate in accordance with people’s lives and needs will be essential. High-quality health systems should maximize people’s health, confidence and economic welfare and do so efficiently and equitably. Investing in high quality primary care that people want to use is a critical first step.
